# Supplementary material for: SMG5-SMG7 authorize nonsense-mediated mRNA decay by enabling SMG6 endonucleolytic activity
Source: Nat Commun. 2021 Jun 25;12:3965. doi: 10.1038/s41467-021-24046-3 (PMC8233366; doi:10.1038/s41467-021-24046-3)
Supplement: Supplementary file 1 — Supplementary Information [file 41467_2021_24046_MOESM1_ESM.pdf]

Supplementary Information

SMG5-SMG7 authorize nonsense-mediated mRNA decay by enabling SMG6 endonucleolytic activity

Volker Boehm[1,2,7,*], Sabrina Kueckelmann[1,2,7], Jennifer V. Gerbracht[1,2], Sebastian Kallabis[3], Thiago Britto-Borges[4,5], Janine Altmüller[6], Marcus Krüger[2,3], Christoph Dieterich[4,5], Niels H. Gehring[1,2,*]

[1] Institute for Genetics, University of Cologne, 50674 Cologne, Germany
[2] Center for Molecular Medicine Cologne (CMMC), University of Cologne, 50937 Cologne, Germany
[3] CECAD Research Center, University of Cologne, Joseph-Stelzmann-Str. 26, 50931 Cologne, Germany
[4] Section of Bioinformatics and Systems Cardiology, Department of Internal Medicine III and Klaus Tschira Institute for Integrative Computational Cardiology, Heidelberg University Hospital, 69120 Heidelberg, Germany
[5] DZHK (German Centre for Cardiovascular Research), Partner site Heidelberg/Mannheim, 69120 Heidelberg, Germany
[6] Cologne Center for Genomics (CCG), University of Cologne, 50931 Cologne, Germany
[7] These authors contributed equally: Volker Boehm, Sabrina Kueckelmann

*Correspondence: boehmv@uni-koeln.de (V.B.), ngehring@uni-koeln.de (N.H.G.)

This PDF file includes:

Extended Data Fig. 1 to 7

Boehm, Kueckelmann et al. 2021: Extended Data Fig. 1

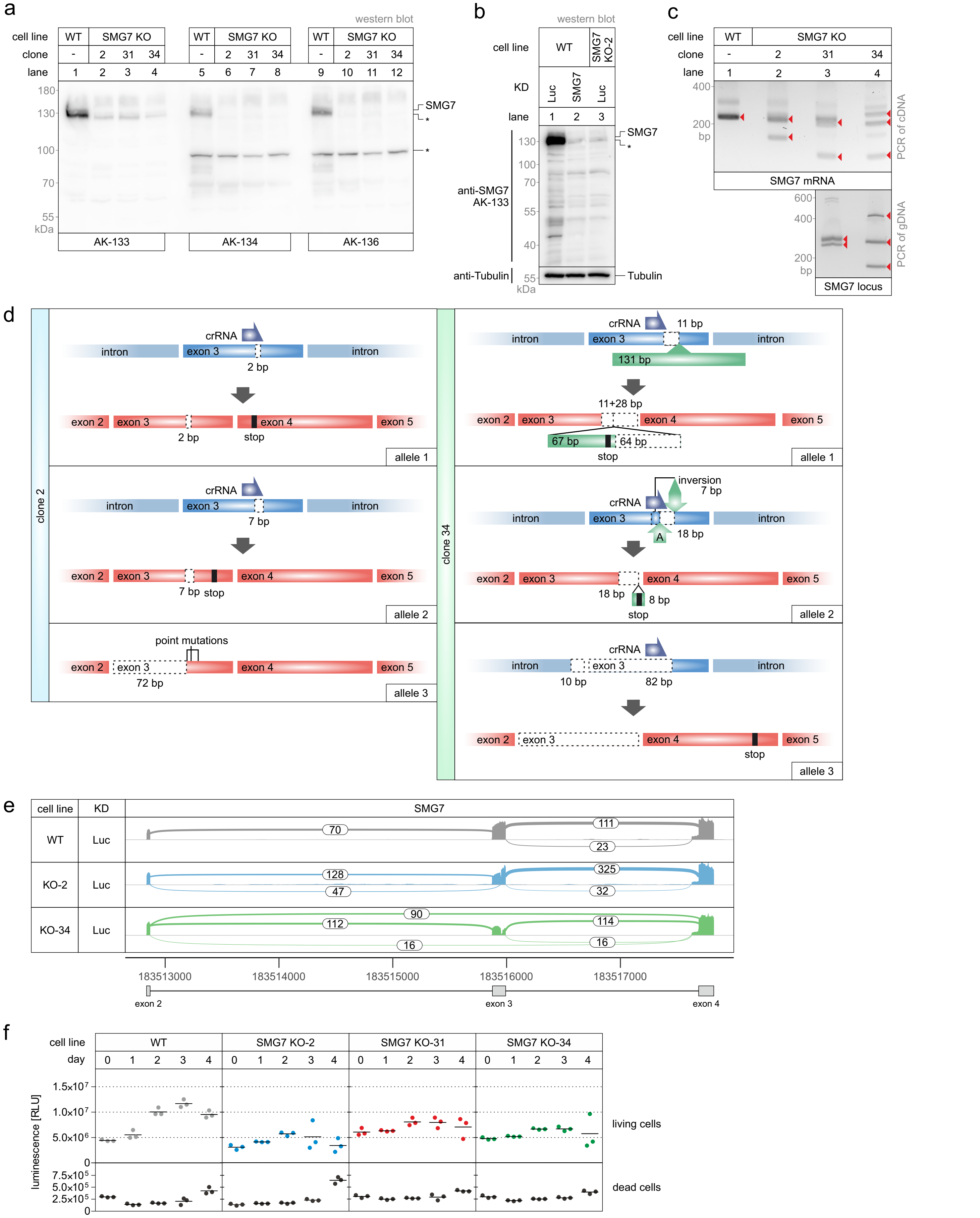

Extended Data Fig. 1: Characterization of SMG7 knockout cells.

a, Uncropped western blot analysis of SMG7 knockout (KO) cell lines as shown in Fig. 1b (n=1). Asterisks indicate non-specific bands.

b, Western blot comparison between SMG7 knockout (KO) and siRNA-mediated knockdown (KD) conditions with AK-133 (n=1). Asterisks indicate non-specific bands.

c, Detection of SMG7 genomic alterations (bottom) and splicing variants (top) via PCR (n=1). Red arrows indicate identified bands.

d, Overview of CRISPR-Cas9 induced alterations on the genomic and transcriptomic level concerning the SMG7 locus.

e, Sashimi plot (via IGV) of the SMG7 splicing pattern in WT, SMG7 KO-2 and KO-34 clones from RNA-Seq data.

f, Luminescence-based growth assay of the indicated cell lines with quantification of live and dead cells at the indicated time points. Individual data points and means are plotted as relative light units [RLU] (n=3 biologically independent samples).

Boehm, Kueckelmann et al. 2021: Extended Data Fig. 2

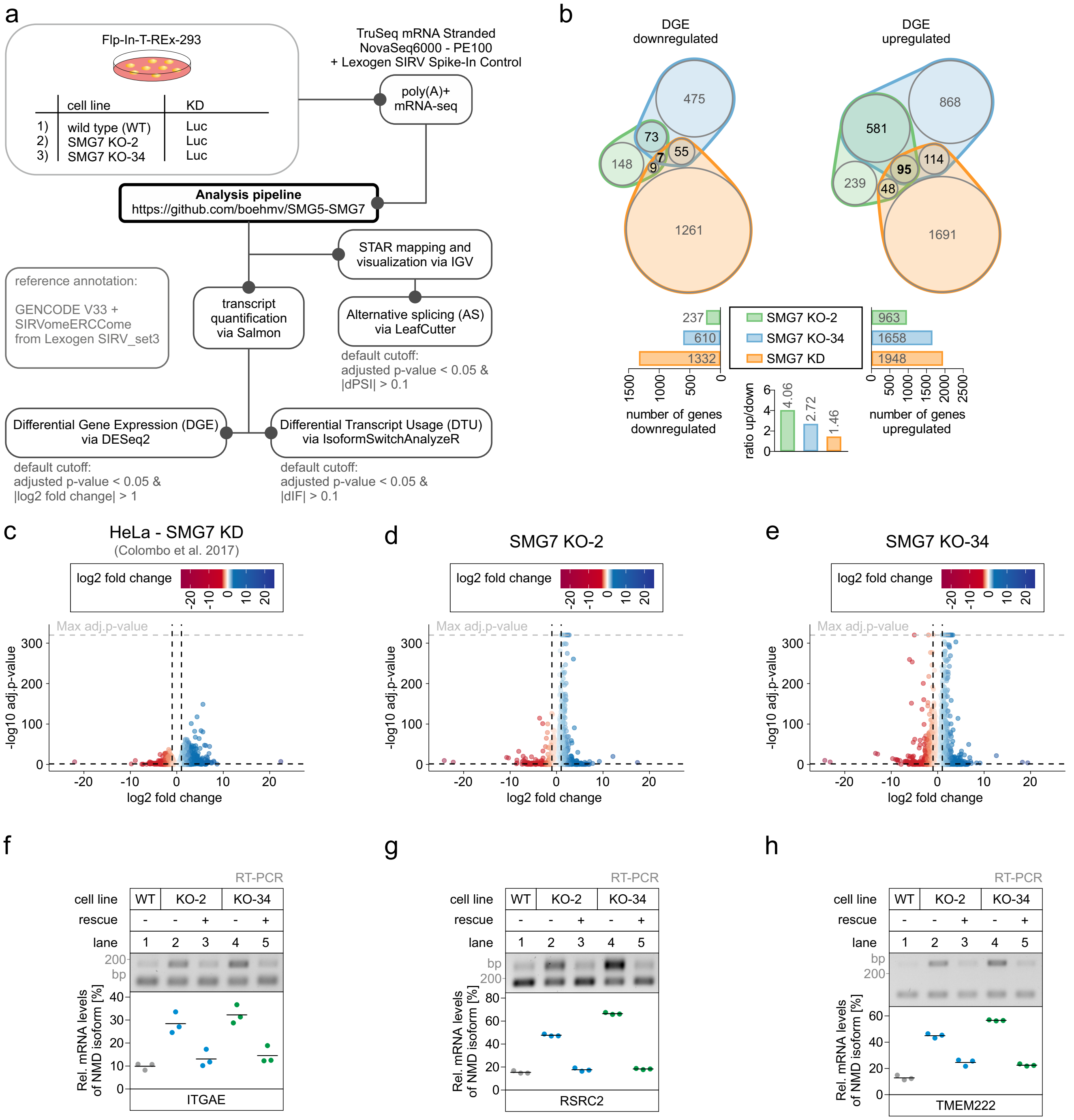

Extended Data Fig. 2: RNA-Seq analyses of SMG7 KO cells.

a, Schematic overview of the samples (n=3 biologically independent samples) and RNA-Seq analysis pipeline. The codes used in this study are available at GitHub [https://github.com/boehmv/SMG5-SMG7].

b, Overlap of upregulated and downregulated genes in the differential gene expression (DGE; via DESeq2) analysis. Total numbers and ratios are given below. Created with the nVenn algorithm.

c-e, Volcano plots showing the differential gene expression analyses from the indicated RNA-Seq datasets. The log2 fold change is plotted against the -log10 adjusted p-value (adj.p-value). P-values were calculated by DESeq2 using a two-sided Wald test and corrected for multiple testing using the Benjamini-Hochberg method.

f-h, End-point RT-PCR detection of ITGAE (**f**), RSRC2 (**g**) and TMEM222 (**h**) transcript isoforms was carried out in the indicated cell lines, with or without expression of FLAG-tagged SMG7 rescue constructs. Data points and means from the gel quantification are plotted (n=3 biologically independent samples).

Boehm, Kueckelmann et al. 2021: Extended Data Fig. 3

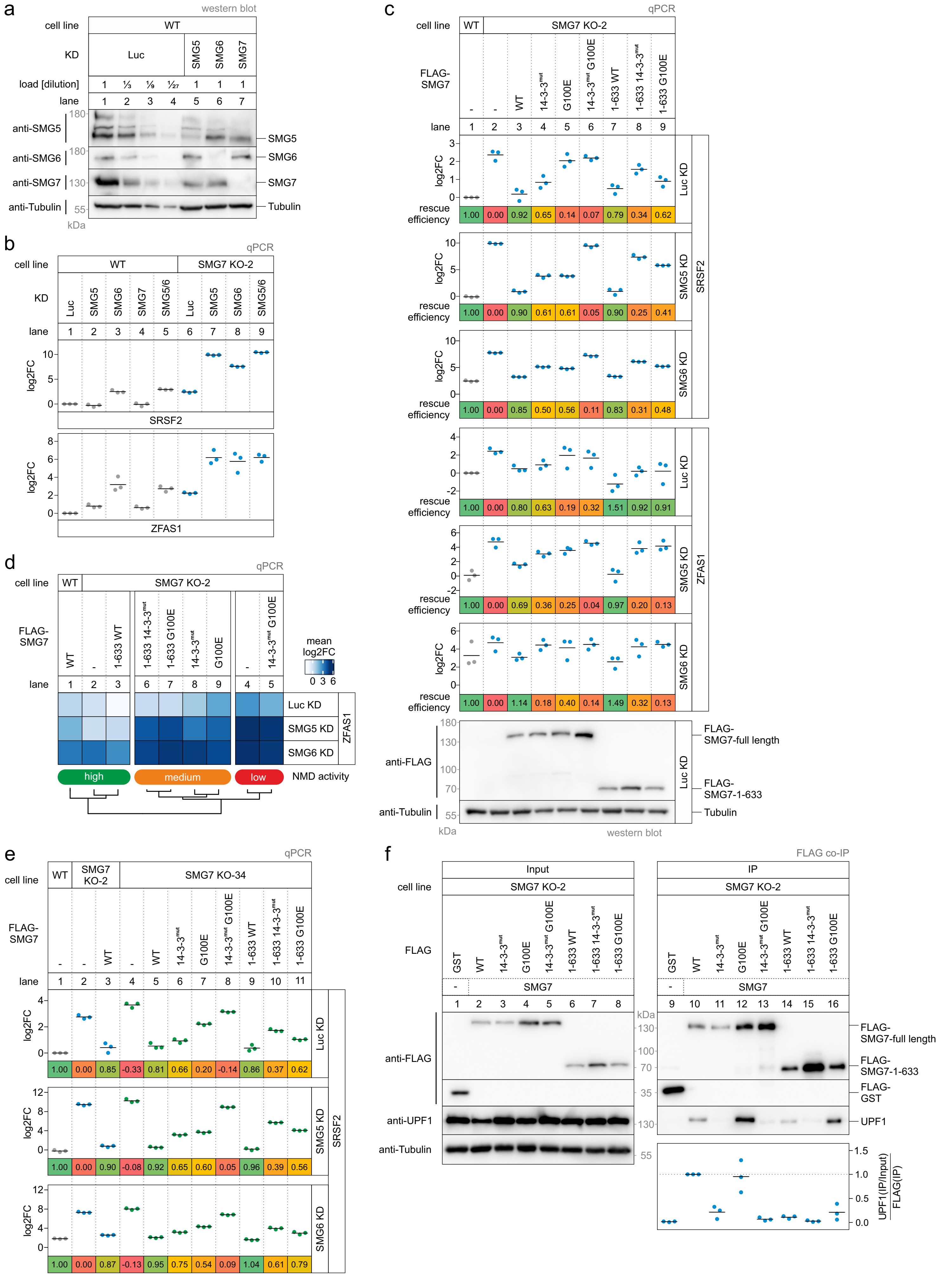

Extended Data Fig. 3: SMG7 supports NMD independently of the deadenylation-promoting function.

Figure legend on next page

Extended Data Fig. 3: SMG7 supports NMD independently of the deadenylation-promoting function.

a, Western blot analysis of individual SMG5, SMG6 and SMG7 KDs in WT cells with a dilution series of control KD samples (n=1). Tubulin serves as control.

b, Quantitative RT-PCR-based detection (qPCR) of SRSF2 isoforms and ZFAS1 in the indicated cell lines upon treatment with the indicated siRNA. The ratio of NMD isoform to canonical isoform (SRSF2) and ZFAS1 to the C1orf43 reference was calculated; mean log2 fold change (log2FC) is shown (n=3 biologically independent samples). The corresponding heatmap is plotted in Fig. 2e.

c, Quantitative RT-PCR-based detection (qPCR) of SRSF2 isoforms in the indicated cell lines upon treatment with the indicated siRNA and expression of the indicated FLAG-tagged rescue constructs. The ratio of NMD isoform to canonical isoform (SRSF2) was calculated; mean log2 fold change (log2FC) is shown (n=3 biologically independent samples). Rescue efficiency was calculated based on the mean log2FC in relation lane 1 (set to 1) and lane 2 (set to 0). The corresponding heatmap is plotted in Fig. 2f. Western blot analyses are shown below (n=1). Tubulin serves as control.

d, Heatmap of quantitative RT-PCR-based detection (qPCR) of ZFAS1 in the indicated cell lines upon treatment with the indicated siRNA and expression of the indicated FLAG-tagged rescue constructs. The ratio of ZFAS1 to the C1orf43 reference was calculated; mean log2 fold change (log2FC) is shown (n=3 biologically independent samples). Clustering (k=3) for NMD activity is depicted below.

e, Quantitative RT-PCR-based detection (qPCR) of SRSF2 isoforms in the indicated cell lines upon treatment with the indicated siRNA and expression of the indicated FLAG-tagged rescue constructs. The ratio of NMD isoform to canonical isoform (SRSF2) was calculated; mean log2 fold change (log2FC) is shown (n=3 biologically independent samples). Rescue efficiency was calculated based on the mean log2FC in relation lane 1 (set to 1) and lane 2 (set to 0).

f, Western blot after FLAG co-immunoprecipitation (IP) of FLAG-tagged GST (control) or SMG7 constructs in SMG7 KO cells. Tubulin serves as control. Quantification results are shown as data points and mean (n=3 biologically independent samples).

Boehm, Kueckelmann et al. 2021: Extended Data Fig. 4

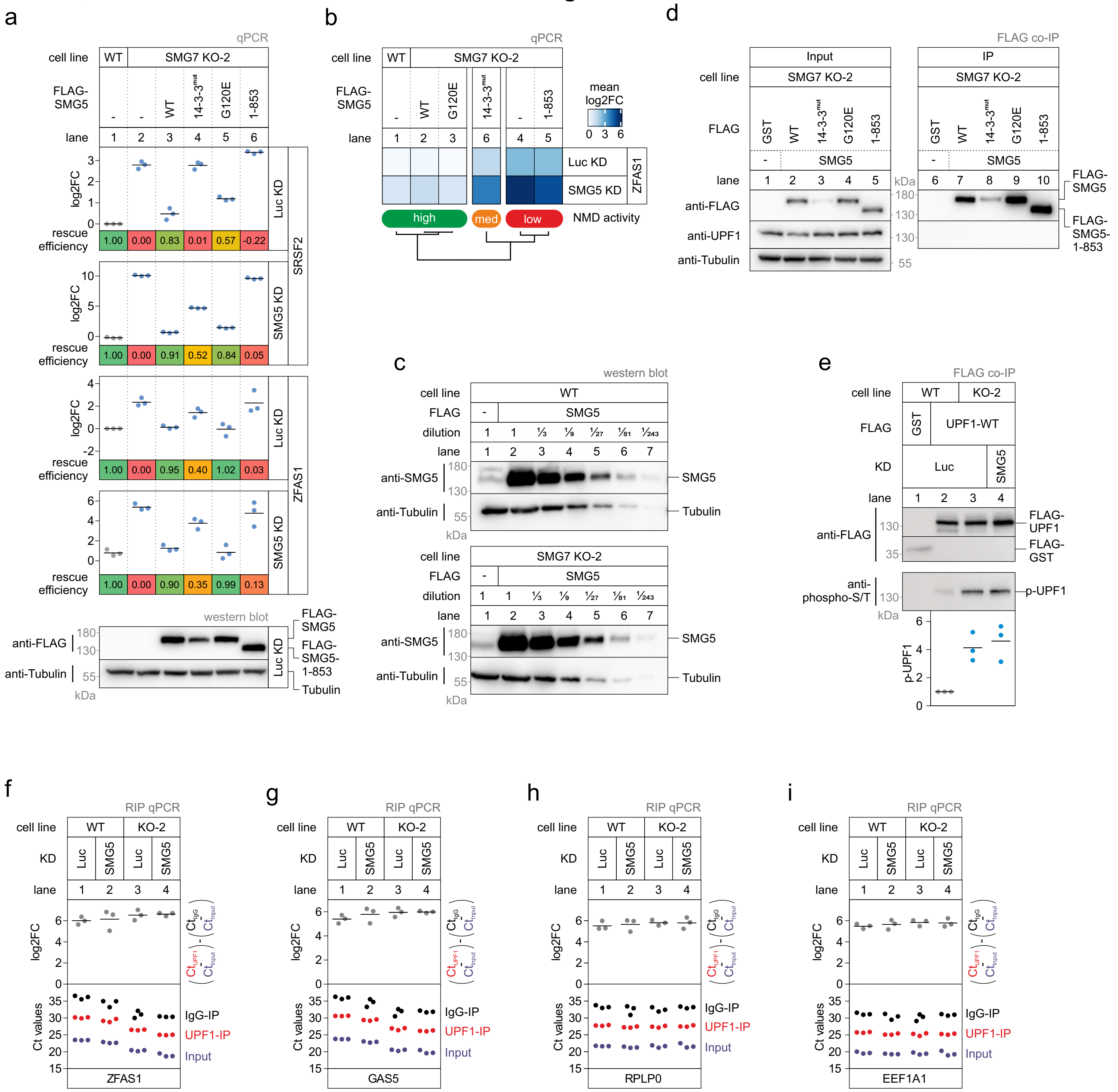

Extended Data Fig. 4: Characterization of SMG5 function in NMD.

a, Quantitative RT-PCR-based detection (qPCR) of SRSF2 isoforms in the indicated cell lines upon treatment with the indicated siRNA and expression of the indicated FLAG-tagged rescue constructs. The ratio of NMD isoform to canonical isoform (SRSF2) was calculated; mean log2 fold change (log2FC) is shown (n=3 biologically independent samples). Rescue efficiency was calculated based on the mean log2FC in relation lane 1 (set to 1) and lane 2 (set to 0). The corresponding heatmap is plotted in Fig. 3b. Western blot analyses are shown below (n=1). Tubulin serves as control.

b, Heatmap of quantitative RT-PCR-based detection (qPCR) of ZFAS1 in the indicated cell lines upon treatment with the indicated siRNA and expression of the indicated FLAG-tagged rescue constructs. The ratio of ZFAS1 to the C1orf43 reference was calculated; mean log2 fold change (log2FC) is shown (n=3 biologically independent samples). Clustering (k=3) for NMD activity is depicted below.

c, Western blot analysis of SMG5 expression levels in WT or SMG7 KO cells upon SMG5 overexpression (with dilution series) compared to the respective control cells (n=1). Tubulin serves as control.

d, Western blot after FLAG co-immunoprecipitation (IP) of FLAG-tagged GST (control) or SMG5 constructs in SMG7 KO cells (n=3 biologically independent samples). Tubulin serves as control.

e, Analysis of UPF1 phosphorylation status after IP of expressed FLAG-tagged UPF1. Quantification results are shown as data points and mean (n=3 biologically independent samples).

f-i, qPCR detection of ZFAS1 (**f**), GAS5 (**g**), RPLP0 (**h**) and EEF1A1 (**i**) was carried out in UPF1-IP, control IgG-IP and Input samples after RNA immunoprecipitation (RIP) from the indicated conditions. Data points and means from the qPCRs are plotted as log2 fold change (log2FC) (n=3 biologically independent samples). Raw Ct values are shown for comparison.

Boehm, Kueckelmann et al. 2021: Extended Data Fig. 5

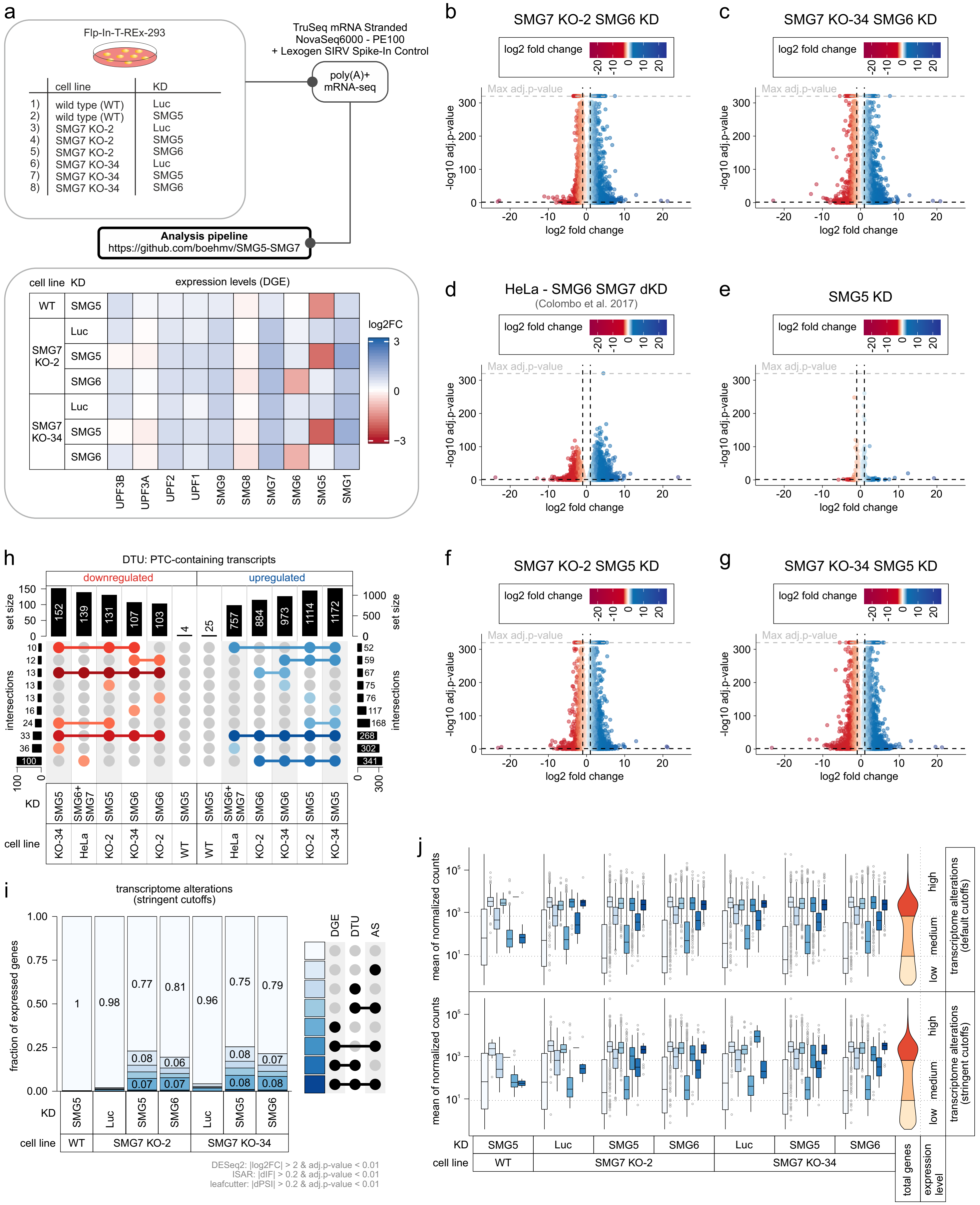

Extended Data Fig. 5: Analyses of SMG7 KO plus knockdown RNA-seq data.

a, Schematic overview of all samples (n=3 biologically independent samples) for the RNA-Seq analysis pipeline (top; same pipeline as depicted in Extended Data Fig. 2a). Differential gene expression analysis (DGE) with DESeq2 of NMD factors is shown at the bottom as heatmap and plotted as log2 fold change (log2FC).

b-g, Volcano plots showing the differential gene expression analyses from the indicated RNA-Seq datasets. The log2 fold change is plotted against the -log10 adjusted p-value (adj.p-value). P-values were calculated by DESeq2 using a two-sided Wald test and corrected for multiple testing using the Benjamini-Hochberg method.

h, Overlap of up- or downregulated premature termination codon (PTC)-containing isoforms in the different RNA-Seq data is shown as UpSet plot. Only the top 10 overlapping sets are shown.

i, Fraction of expressed genes (genes with non-zero counts in DESeq2) were calculated which exhibit individual or combinations of differential gene expression (DGE), differential transcript usage (DTU) and/or alternative splicing (AS) events in the indicated conditions using the respective computational analysis (stringent cutoffs are indicated). AS and DTU events were collapsed on the gene level. For DGE, p-values were calculated by DESeq2 using a two-sided Wald test and corrected for multiple testing using the Benjamini-Hochberg method. For DTU, p-values were calculated by IsoformSwitchAnalyzeR using a DEXSeq-based test and corrected for multiple testing using the Benjamini-Hochberg method. For AS, p-values were calculated by LeafCutter using an asymptotic Chi-squared distribution and corrected for multiple testing using the Benjamini-Hochberg method.

j, Boxplots showing the distribution of different combinations of transcriptomic alteration events in relation to the expression of the gene (indicated by the mean of the normalized counts), with default (top) or stringent (bottom) cutoffs (n=3 biologically independent samples). The center line represents the 50th percentile (median), whereas the lower and upper box limits correspond to the 25th and 75th percentiles. The whiskers extend from the box limits to the smallest or largest value no further than 1.5 * inter-quartile range. Data beyond the end of the whiskers are plotted individually.The total distribution of gene expression is shown on the right as violin plot. Colour coding as in Extended Data Fig. 5i.

Boehm, Kueckelmann et al. 2021: Extended Data Fig. 6

a

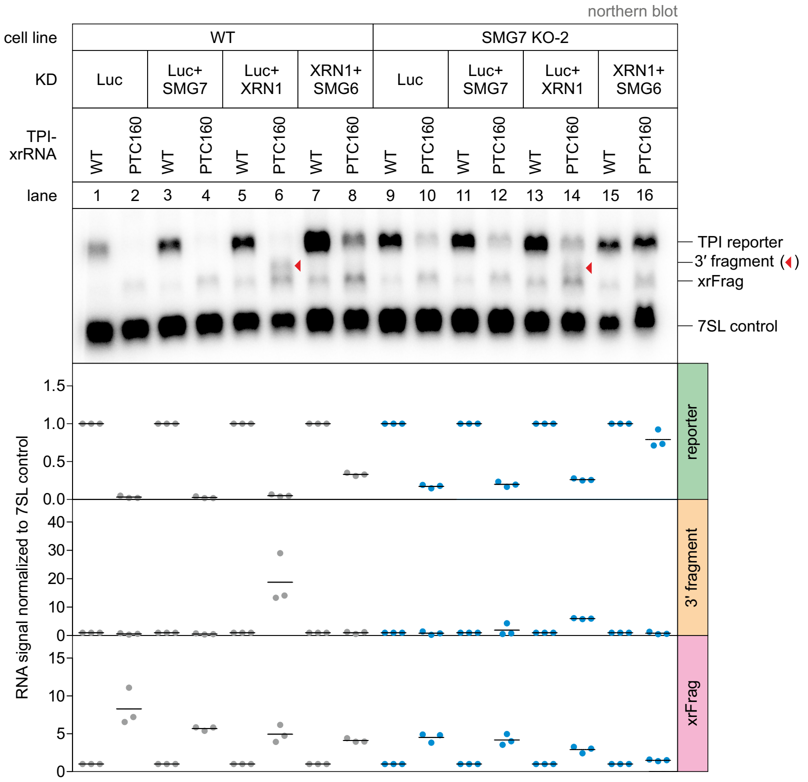

b

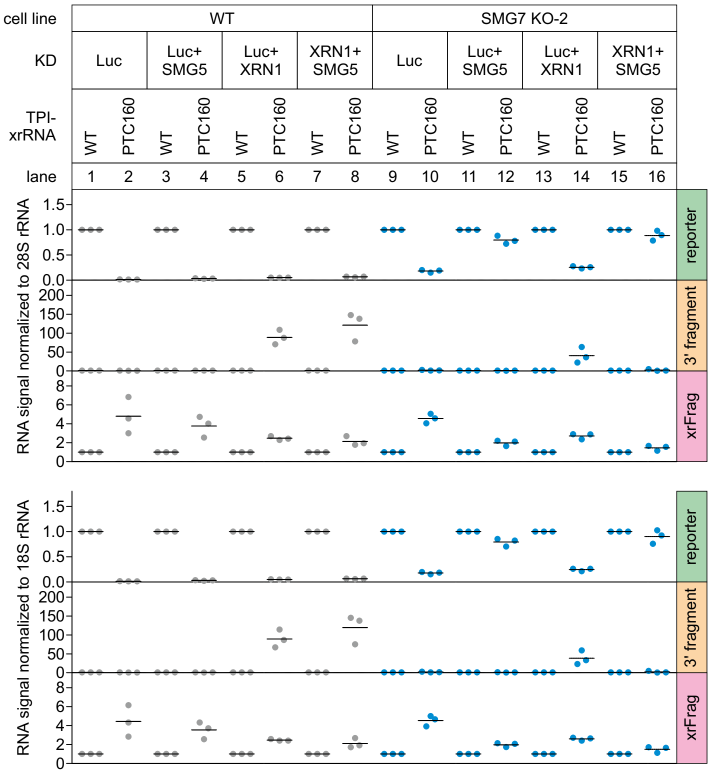

Extended Data Fig. 6: Northern blot analysis of endonucleolytic cleavage.

a, Northern blot analysis of TPI reporter, 3′ fragments (indicated with red triangles), xrFrag and 7SL endogenous control. Ethidium bromide stained 28S and 18S rRNAs are shown as additional controls. Quantification results (normalized to 7SL control) are shown as data points and mean (n=3 biologically independent samples).

b, Quantification of Fig. 5b with 28S or 18S rRNA as reference. Quantification results are shown as data points and mean (n=3 biologically independent samples).

Boehm, Kueckelmann et al. 2021: Extended Data Fig. 7

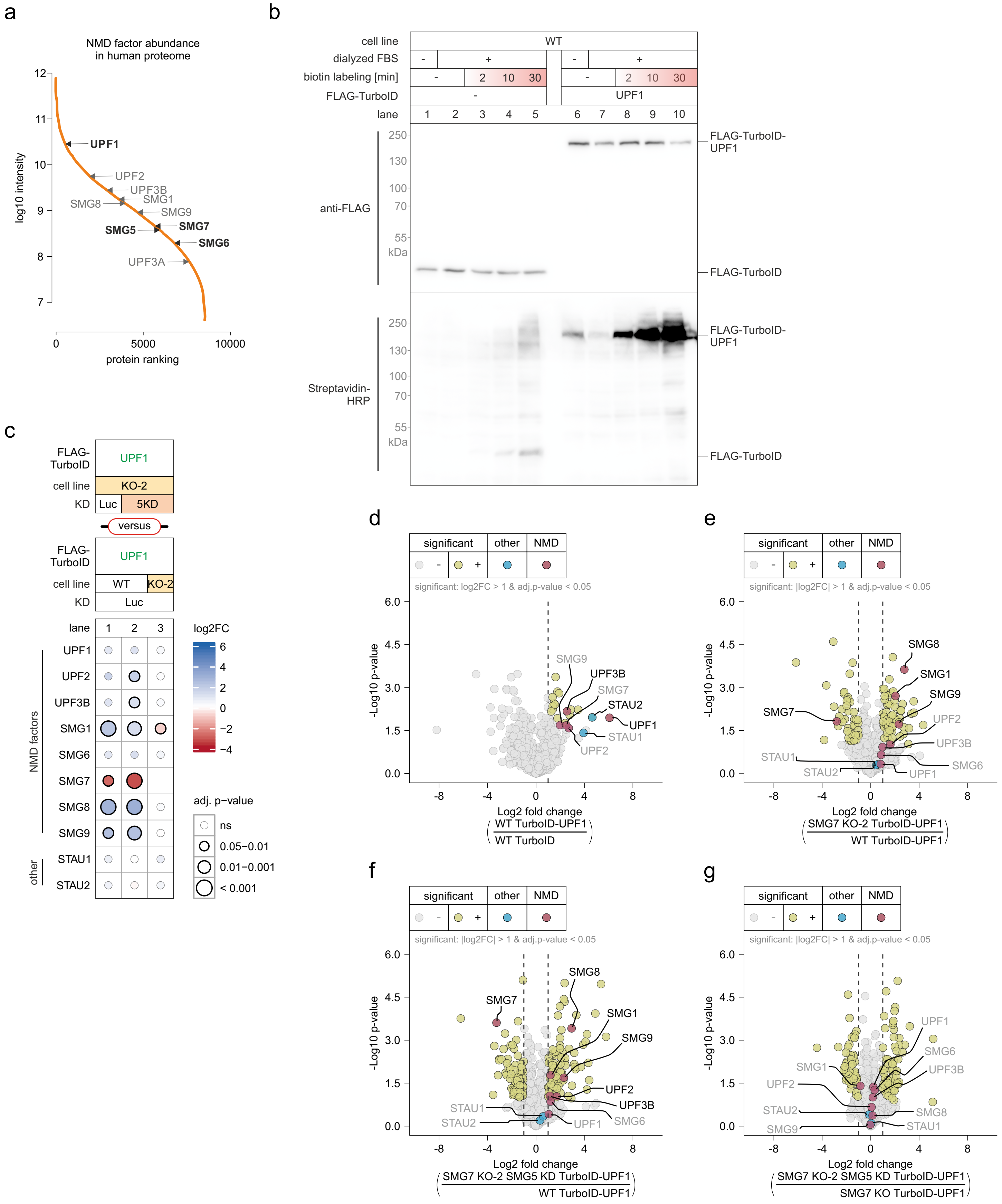

Extended Data Fig. 7: Proximity labelling with FLAG-TurboID-UPF1.

a, Protein abundance distribution of NMD factors in humans, showing the respective intensities (log10 transformed) and protein rank (DOI: 10.1038/s41586-020-2402-x). Data were retrieved from https://proteomesoflife.org/ with the human taxonomy identifier (9606) on 2021-03-03 (03. March 2021).

b, Time-dependent activity test of FLAG-TurboID constructs in WT cells with (+) or without (-) the pre-incubation in medium with dialyzed FBS, followed by western blotting (n=1).

c, Heatmap of mass spectrometry-based analysis of streptavidin-enriched biotinylated NMD and selected other proteins in the respective comparison of conditions (n=3 biologically independent samples). Coloured points indicate the log2 fold change (log2FC) and point size corresponds to the adjusted p-value (adj. p-value; from two-sided Welch's t-test).

d-g, Volcano plots of mass spectrometry-based analysis of streptavidin-enriched biotinylated proteins in the respective comparison of conditions (n=3 biologically independent samples). (**d**) FLAG-TurboID-UPF1 against FLAG-TurboID control in WT cells, (**e**) FLAG-TurboID-UPF1 in SMG7 KO cells against FLAG-TurboID-UPF1 in WT cells, (**f**) FLAG-TurboID-UPF1 in SMG7 KO + SMG5 KD cells against FLAG-TurboID-UPF1 in WT cells, (**g**) FLAG-TurboID-UPF1 in SMG7 KO + SMG5 KD cells against FLAG-TurboID-UPF1 in SMG7 KO cells. The yellow colour labelling indicates targets that are significant in the respective comparisons after two-sided Welch's t-testing (log2 fold change (log2FC) >1 or |log2FC| >1; and adj. p-value < 0.05). Points labelled in blue indicate other proteins of interest; points labelled in red indicate NMD factors. Highlighted proteins that were not significant in the respective comparisons are labelled with grey text.
