# Supplementary material for: SMG5-SMG7 authorize nonsense-mediated mRNA decay by enabling SMG6 endonucleolytic activity
Source: Nat Commun. 2021 Jun 25;12:3965. doi: 10.1038/s41467-021-24046-3 (PMC8233366; doi:10.1038/s41467-021-24046-3)
Supplement: Supplementary file 9 — Description of additional supplementary files [file 41467_2021_24046_MOESM9_ESM.docx]

Description of additional supplementary information

Title: Supplementary Data 1

Description: Differential gene expression (DGE) analysis of SMG7 KO + different KD RNA-seq data using DESeq2

Title: Supplementary Data 2

Description: Alternative splicing (AS) analysis of SMG7 KO + different KD RNA-seq data using LeafCutter

Title: Supplementary Data 3

Description: Differential transcript usage (DTU) analysis of SMG7 KO + different KD RNA-seq data using IsoformSwitchAnalyzeR

Title: Supplementary Data 4

Description: Label-free mass spectrometry analysis after coimmunoprecipitation of FLAG-tagged control or SMG5 proteins

Title: Supplementary Data 5

Description: Label-free mass spectrometry analysis after streptavidin-enrichment of biotinylated proteins in TurboID-control or -UPF1 expressing cell lines

Title: Supplementary Data 6

Description: List of cell lines, plasmids, antibodies, primers and siRNAs used in this study
