# Supplementary material for: SMG5-SMG7 authorize nonsense-mediated mRNA decay by enabling SMG6 endonucleolytic activity
Source: Nat Commun. 2021 Jun 25;12:3965. doi: 10.1038/s41467-021-24046-3 (PMC8233366; doi:10.1038/s41467-021-24046-3)
Supplement: Supplementary file 10 — Reporting Summary [file 41467_2021_24046_MOESM10_ESM.pdf]

nature research

Corresponding author(s): Volker Boehm and Niels H. Gehring

Last updated by author(s): May 14, 2021

Reporting Summary

Nature Research wishes to improve the reproducibility of the work that we publish. This form provides structure for consistency and transparency in reporting. For further information on Nature Research policies, see our Editorial Policies and the Editorial Policy Checklist.

Statistics

For all statistical analyses, confirm that the following items are present in the figure legend, table legend, main text, or Methods section.

n/a	Confirmed	
☐	☒	The exact sample size (*n*) for each experimental group/condition, given as a discrete number and unit of measurement
☐	☒	A statement on whether measurements were taken from distinct samples or whether the same sample was measured repeatedly
☐	☒	The statistical test(s) used AND whether they are one- or two-sided<br>*Only common tests should be described solely by name; describe more complex techniques in the Methods section.*
☒	☐	A description of all covariates tested
☒	☐	A description of any assumptions or corrections, such as tests of normality and adjustment for multiple comparisons
☐	☒	A full description of the statistical parameters including central tendency (e.g. means) or other basic estimates (e.g. regression coefficient) AND variation (e.g. standard deviation) or associated estimates of uncertainty (e.g. confidence intervals)
☐	☒	For null hypothesis testing, the test statistic (e.g. *F*, *t*, *r*) with confidence intervals, effect sizes, degrees of freedom and *P* value noted<br>*Give P values as exact values whenever suitable.*
☒	☐	For Bayesian analysis, information on the choice of priors and Markov chain Monte Carlo settings
☒	☐	For hierarchical and complex designs, identification of the appropriate level for tests and full reporting of outcomes
☒	☐	Estimates of effect sizes (e.g. Cohen's *d*, Pearson's *r*), indicating how they were calculated

Our web collection on statistics for biologists contains articles on many of the points above.

Software and code

Policy information about availability of computer code

Data collection	Protein bands detected with the Fusion FX-6 Edge system (Vilber Lourmat) using the Evolution-Capt Edge software (version 18.05) were quantified in a semi-automated manner using the ImageQuant TL 1D software (version 8.1) with a rolling-ball background correction. The luminometer centro XS3 LB 960 (Berthold Technologies) and the MikroWin software (version 5.14) was used for collecting growth assay data. The Gel Doc XR+ (Bio-Rad) and Image Lab software (version 5.1) was used to visualize EtBr- or TCE-stained gels. The CFX96 Touch Real-Time PCR Detection System (Bio-Rad) with Bio-Rad CFX Manager software (version 3.0) was used for quantitative RT-PCR. Northern blots were imaged using the Typhoon FLA 7000 (GE Healthcare). The RNA-Seq reads were generated with an Illumina NovaSeq6000 sequencing instrument and a PE100 protocol. Proteomics analysis was performed by data-dependent acquisition using an Easy nLC1000 ultra high-performance liquid chromatography (UHPLC) system coupled via nanoelectrospray ionization to a Q Exactive Plus instrument (all Thermo Scientific). Details for RNA-Seq and mass spectrometry data collection can be found in the Methods section.

Data analysis

A comprehensive description of the used computer code for the RNA-Seq analysis is available at GitHub [https://github.com/boehmv/SMG5-SMG7] and Zenodo [https://doi.org/10.5281/zenodo.4603388]. RNA-Seq reads were aligned against the human genome (version 38, GENCODE release 33 transcript annotations supplemented with SIRVomeERCCome annotations from Lexogen) using the STAR read aligner (version 2.7.3a). Transcript abundance estimates were computed with Salmon (version 1.3.0) with a decoy-aware transcriptome. Differential gene expression analysis was performed with the DESeq2 R package (version 1.28.1). Differential splicing was detected with LeafCutter (version 0.2.9). Differential transcript usage was computed with IsoformSwitchAnalyzeR (version 1.10.0). Integrative Genomics Viewer (IGV) (version 2.8.12) snapshots were generated from mapped reads (BAM files). For proteomics, MS RAW files were analysed using the standard settings of the MaxQuant suite (version 1.6.17.0). Data processing and statistical analysis was done in the Perseus software (version 1.6.5.0). The analysis of mass spectrometry data is explained in the Methods section in detail. Quantifications and calculations for other experiments were performed - if not indicated otherwise - with Microsoft Excel (version 1808) or R (version 4.0.4) and all plots were generated using IGV (version 2.8.12), GraphPad Prism 5, ggplot2 (version 3.3.3) or ComplexHeatmap (version 2.6.2). Protein structures were superimposed using Chimera (version 1.13) and visualized using ChimeraX (version 1.0).

For manuscripts utilizing custom algorithms or software that are central to the research but not yet described in published literature, software must be made available to editors and reviewers. We strongly encourage code deposition in a community repository (e.g. GitHub). See the Nature Research guidelines for submitting code & software for further information.

Data

Policy information about availability of data

All manuscripts must include a data availability statement. This statement should provide the following information, where applicable:

- Accession codes, unique identifiers, or web links for publicly available datasets
- A list of figures that have associated raw data
- A description of any restrictions on data availability

RNA-sequencing data generated for this manuscript have been deposited in the ArrayExpress database at EMBL-EBI (www.ebi.ac.uk/arrayexpress) under accession number E-MTAB-9330 [https://www.ebi.ac.uk/arrayexpress/experiments/E-MTAB-9330]. Published datasets analysed for this paper include: Gene Expression Omnibus (GEO) accession number GSE86148 [https://www.ncbi.nlm.nih.gov/geo/query/acc.cgi?acc=GSE86148]. Data of human proteome abundances were retrieved from https://proteomesoflife.org/ with the human taxonomy identifier (9606) on 2021-03-03 (03. March 2021). The mass spectrometry proteomics data have been deposited to the ProteomeXchange Consortium via the PRIDE partner repository with the dataset identifier PXD024747 [https://www.ebi.ac.uk/pride/archive/projects/PXD024747]. Published protein structures were human SMG7 (PDB: 1YA0 [http://doi.org/10.2210/pdb1YA0/pdb]) and C. elegans SMG5-SMG7 (PDB: 3ZHE [http://doi.org/10.2210/pdb3ZHE/pdb]). All relevant data supporting the key findings of this study are available within the article and its Supplementary Information files or from the corresponding author upon reasonable request. A reporting summary for this Article is available as a Supplementary Information file. Source Data – where applicable - are provided for all figures, including raw images of EtBr-stained agarose gels, western blots and northern blots as well as qPCR raw values, quantifications and an overview file stating all further necessary information (e.g. which antibody was used). All raw source data can be accessed at Zenodo [https://doi.org/10.5281/zenodo.4603279].

Field-specific reporting

Please select the one below that is the best fit for your research. If you are not sure, read the appropriate sections before making your selection.

☒ Life sciences ☐ Behavioural & social sciences ☐ Ecological, evolutionary & environmental sciences

For a reference copy of the document with all sections, see nature.com/documents/nr-reporting-summary-flat.pdf

Life sciences study design

All studies must disclose on these points even when the disclosure is negative.

Sample size

No statistical methods were performed to predetermine sample size. The numbers of replicates (=sample size) are given in the Figure Legend for each experiment. For most experiments this sample size was three independent biological replicates, as is standard for similar molecular biology experiments. Comparable sample sizes were chosen that allow data reproducibility for each experimental conditions. For all data - if possible - positive and negative controls were included.

Data exclusions

No data exclusion criteria were pre-established besides default significance and effect cutoffs in analyses such as RNA-sequencing (values described in the manuscript), which are according to standards in the field. These default cutoffs were: Differential gene expression |log2FoldChange|> 1 and adjusted p-value (padj) < 0.05; Differential splicing |deltapsi| > 0.1 and adjusted p-value (p.adjust) < 0.05; Differential transcript usage |dIF| > 0.1 and adjusted p-value (isoform_switch_q_value) < 0.05

Replication

RNA-sequencing runs were performed once with triplicates of distinct samples for each condition.
Quantified end-point PCR, qPCR, and northern blot experiments were performed at least with triplicates of distinct samples for each condition.
Quantified Western blot analysis were performed at least with triplicates of distinct samples for each condition.
All attempts at replication were successful.

Randomization

The samples were not randomized in this study, since we did not perform experiments or statistical analyses that require randomization.

Blinding

The investigators were not blinded during data collection because the experiments did not require blinding and were readily controlled without blinding. Investigators were not blinded during data analyses because the key findings are supported by quantitative measurements (with statistical testing where relevant) that do not rely heavily on subjective judgment for interpretation.

Reporting for specific materials, systems and methods

We require information from authors about some types of materials, experimental systems and methods used in many studies. Here, indicate whether each material, system or method listed is relevant to your study. If you are not sure if a list item applies to your research, read the appropriate section before selecting a response.

Materials & experimental systems

n/a	Involved in the study
☐	☒ Antibodies
☐	☒ Eukaryotic cell lines
☒	☐ Palaeontology and archaeology
☒	☐ Animals and other organisms
☒	☐ Human research participants
☒	☐ Clinical data
☒	☐ Dual use research of concern

Methods

n/a	Involved in the study
☒	☐ ChIP-seq
☒	☐ Flow cytometry
☒	☐ MRI-based neuroimaging

Antibodies

Antibodies used

Description in the following order: Name | Dilution | Source
Mouse monoclonal anti-alpha-Tubulin (clone B-5-1-2) | 1:5000 | Sigma-Aldrich; Cat# T6074; RRID:AB_477582
Mouse monoclonal anti-FLAG (clone M2) | 1:3000 | Sigma-Aldrich Cat# F3165, RRID:AB_259529
Rabbit polyclonal anti-FLAG | 1:3000 | Sigma-Aldrich; Cat# F7425; RRID:AB_439687
Rabbit monoclonal anti-FLAG (clone D6W5B) | 1:2000 | Cell Signaling Technology; Cat# 14793; RRID:AB_2572291
Rabbit polyclonal anti-SMG5 | 1:1000 | Proteintech; Cat# 12694-1-AP; RRID:AB_2270781
Rabbit polyclonal anti-SMG6 | 1:3000 | Abcam; Cat# ab87539; RRID:AB_10674461
Rabbit polyclonal anti-SMG7 | 1:2000 | Bethyl; Cat# A302-170A; RRID:AB_1659842
Rabbit polyclonal anti-SMG7 | 1:2000 | Elabscience; Cat# E-AB-32926; RRID: N/A
Rabbit polyclonal anti-SMG7 | 1:1000 | Biorbyt; Cat# orb214942-30; RRID: N/A
Rabbit polyclonal anti-phospho-Upf1 (Ser1127) | 1:1000 | Millipore Cat# 07-1016, RRID:AB_10805931
Rabbit polyclonal anti-UPF3B | 1:2000 | Eurogentech, custom-made, raised against C-terminal UPF3B (300-483) and affinity purified
Goat polyclonal anti-UPF1 | 1:1000 | Bethyl; Cat# A300-038A-M; RRID:AB_2778977
Rabbit monoclonal anti-Phospho-(Ser/Thr) ((clone 4F7) | 1:3000 | Cell Signaling Technology; Cat# 2909S; RRID:AB_2163443
Purified Goat IgG | 5 µg for RIP | Bethyl Cat# P50-100, RRID:AB_479842
Goat polyclonal anti-UPF1 | 5 µg for RIP | Bethyl Cat# A300-036A, RRID:AB_203272
Streptavidin-HRP | 1:2000 | Cellsignaling Cat# 3999, RRID:AB_10830897
Peroxidase-AffiniPure Goat Anti-Rabbit | 1:3000 | Jackson ImmunoResearch Labs; Cat# 111-035-006; RRID:AB_2337936
Peroxidase-AffiniPure Goat Anti-Mouse | 1:3000 | Jackson ImmunoResearch Labs; Cat# 115-035-003; RRID:AB_10015289
Peroxidase-AffiniPure Donkey Anti-Goat | 1:3000 | Jackson ImmunoResearch Labs; Cat# 705-035-147; RRID:AB_2313587

Validation

All primary antibodies were recommended or validated by the manufacturer for western blotting and have been tested with suitable overexpression, knockdown, or knockout samples.
Validation statements from the manufacturer (datasheet or homepage):
Mouse monoclonal anti-alpha-Tubulin (clone B-5-1-2) | 1:5000 | Sigma-Aldrich; Cat# T6074; RRID:AB_477582; application(s): western blot: 0.25-0.5 µg/mL using total cell extract of human foreskin fibroblast cell line (FS11); species reactivity: chicken, kangaroo rat, sea urchin, rat, Chlamydomonas, bovine, human, African green monkey, mouse
Mouse monoclonal anti-FLAG (clone M2) | 1:3000 | Sigma-Aldrich Cat# F3165, RRID:AB_259529; application(s): western blot: 10 µg/mL (Protein A); species reactivity: all
Rabbit polyclonal anti-FLAG | 1:3000 | Sigma-Aldrich; Cat# F7425; RRID:AB_439687; application(s): western blot (chemiluminescent): 1-2.5 µg/mL using an E. coli periplasmic extract expressing an N-terminal FLAG fusion protein; species reactivity: all
Rabbit monoclonal anti-FLAG (clone D6W5B) | 1:2000 | Cell Signaling Technology; Cat# 14793; RRID:AB_2572291; Application: WB, IP, IHC-P, IF-IC, F, ChIP; species reactivity: all
Rabbit polyclonal anti-SMG5 | 1:1000 | Proteintech; Cat# 12694-1-AP; RRID:AB_2270781; 12694-1-AP targets SMG5 in WB,ELISA applications and shows reactivity with human samples.
Rabbit polyclonal anti-SMG6 | 1:3000 | Abcam; Cat# ab87539; RRID:AB_10674461; Suitable for: WB, ICC; Reacts with: Human
Rabbit polyclonal anti-SMG7 | 1:2000 | Bethyl; Cat# A302-170A; RRID:AB_1659842; Applications: WB, IP; Reactivity: Human, Mouse
Rabbit polyclonal anti-SMG7 | 1:2000 | Elabscience; Cat# E-AB-32926; RRID: N/A; Applications: WB, IHC-p, IF, ELISA; Reactivity: Human
Rabbit polyclonal anti-SMG7 | 1:1000 | Biorbyt; Cat# orb214942-30; RRID: N/A; Tested Applications: ICC, IF, IHC-P, IP, WB; Reacitvity: Human
Rabbit polyclonal anti-phospho-Upf1 (Ser1127) | 1:1000 | Millipore Cat# 07-1016, RRID:AB_10805931; Anti-phospho-Upf1 (Ser1127), Cat. No. 07-1016, is a highly specific rabbit polyclonal antibody that targets Regulator of nonsense transcripts 1 (RENT1) phosphorylated at Ser1127 and has been tested in Immunoprecipitation, peptide Inhibition Assay, and Western Blotting. Key Applications: WB, IP; Species Reactivity: H, M
Goat polyclonal anti-UPF1 | 1:1000 | Bethyl; Cat# A300-038A-M; RRID:AB_2778977; Applications: WB, IP; Reactivity: Human, Mouse
Rabbit monoclonal anti-Phospho-(Ser/Thr) ((clone 4F7) | 1:3000 | Cell Signaling Technology; Cat# 2909S; RRID:AB_2163443; Phospho-(Ser/Thr) ATM/ATR Substrate (4F7) Rabbit mAb detects endogenous levels of proteins containing the ATM/ATR substrate motif. This antibody preferentially binds peptides and proteins that contain phospho-Ser/Thr preceded by Leu or similar hydrophobic amino acids at the -1 position and followed by Gln at the +1 position. The antibody does not cross-react with corresponding

nonphosphorylated sequences or with other phospho-Ser/Thr-containing motifs. Species Reactivity: All Species Expected
Goat polyclonal anti-UPF1 | 5 µg for RIP | Bethyl Cat# A300-036A, RRID:AB_203272; Applications: WB; Reactivity: Human, Mouse
Streptavidin-HRP | 1:2000 | Cellsignaling Cat# 3999, RRID:AB_10830897; Applications: WB; Reactivity: all

Eukaryotic cell lines

Policy information about cell lines

Cell line source(s)	Parental Flp-In-T-REx-293 cells were purchased from Thermo Fisher Scientific; Cat# R78007; RRID:CVCL_U427<br>All other cell lines were derived from this parental cell line by either usage of the Alt-R CRISPR-Cas9 system (Integrated DNA Technologies) and/or stable integration of derivatives of pcDNA5/FRT/TO vector (Thermo Fisher Scientific) or PB-CuO-MCS-IRES-GFP-EF1α-CymR-Puro vector (System Biosciences). An overview of the used and generated cell lines is given in Supplementary Data 6.
Authentication	The parental Flp-In-T-REx-293 cell line was not further authenticated. SMG7 knockout cell lines were authenticated by western blotting, PCR of cDNA and genomic DNA, and RNA-sequencing (clone 2 and 34). Generated stable cell lines were authenticated by western blotting and/or northern blotting to detect the expressed protein/RNA.
Mycoplasma contamination	The parental cell lines was routinely tested and was free of mycoplasma contamination. Subsequent cell lines were not re-tested during the course of this study.
Commonly misidentified lines<br>(See ICLAC register)	No commonly misidentified cell lines were used in the study.
